# Novel *CNNM2* Mutation Responsible for Autosomal-Dominant Hypomagnesemia With Seizure

**DOI:** 10.3389/fgene.2022.875013

**Published:** 2022-06-29

**Authors:** Min-Hua Tseng, Sung-Sen Yang, Chih-Chien Sung, Jhao-Jhuang Ding, Yu-Juei Hsu, Shih-Ming Chu, Shih-Hua Lin

**Affiliations:** ^1^ Division of Nephrology, Department of Pediatrics, Chang Gung Memorial Hospital and Chang Gung University, Taoyuan, Taiwan; ^2^ Department of Pediatrics, Xiamen Chang Gung Hospital, Ximen, China; ^3^ Division of Nephrology, Department of Internal Medicine, Tri-Service General Hospital, National Defense Medical Center, Taipei, Taiwan; ^4^ Department of Pediatrics, Tri-Service General Hospital, National Defense Medical Center, Taipei, Taiwan; ^5^ Division of Neonatology, Department of Pediatrics, Chang Gung Memorial Hospital and Chang Gung University, Taoyuan, Taiwan

**Keywords:** CNNM2, hypomagnesemia, seizure, HSMR syndrome, renal magnesium wasting

## Abstract

*CNNM2* is primarily expressed in the brain and distal convoluted tubule (DCT) of the kidney. Mutations in *CNNM2* have been reported to cause hypomagnesemia, seizure, and intellectual disability (HSMR) syndrome. However, the clinical and functional effect of CNNM2 mutations remains incompletely understood. We report our clinical encounter with a 1-year-old infant with HSMR features. Mutation screening for this trio family was performed using next-generation sequencing (NGS)-based whole exome sequencing (WES) with the identified mutation verified by Sanger sequencing. We identified a *de novo* heterozygous mutation c.G1439T (R480L) in the essential cystathionine β-synthase (CBS) domain of *CNNM2* encoding CNNM2 (cyclin M2) without any other gene mutations related to hypomagnesemia. The amino acid involved in this missense mutation was conserved in different species. It was also found to be pathogenic based on the different software prediction models and ACGME criteria. *In vitro* studies revealed a higher expression of the CNNM2-R480L mutant protein compared to that of the wild-type CNNM2. Like the CNNM2-wild type, proper localization of CNNM2-R480L was shown on immunocytochemistry images. The Mg^2+^ efflux assay in murine DCT (mDCT) cells revealed a significant increase in intracellular Mg^2+^ green in CNNM2-R480L compared to that in CNNM2-WT. By using a simulation model, we illustrate that the R480L mutation impaired the interaction between CNNM2 and ATP-Mg^2+^. We propose that this novel R480L mutation in the *CNNM2* gene led to impaired binding between Mg^2+^-ATP and CNNM2 and diminished Mg^2+^ efflux, manifesting clinically as refractory hypomagnesemia.

## Introduction

Magnesium (Mg^2+^) is a pivotal cation and cofactor in maintaining cell function (Li et al., 2011; Jahnen-Dechent and Ketteler, 2012; de Baaij et al., 2015). In the gastrointestinal system, it is reabsorbed in the intestine and in the colon by fine-tuned control of an active transcellular Mg^2+^ channel, followed by extrusion to the blood *via* the CNNM4 Na^+^/Mg^2+^ exchanger. In the kidney, most of the filtered Mg^2+^ is reabsorbed paracellularly in the proximal tubule (PT) and thick-ascending loop of Henle (TALH) *via* different claudins (claudins 1 and 2 in the PT, and claudins 10, 14, 16, and 19 in TALH) (Stuiver et al., 2011; de Baaij et al., 2012; Curry and Yu, 2018; Ellison et al., 2021). Like the colon, the distal convoluted tubule (DCT) fine-tunes the reabsorption of tubular Mg^2+^ *via* the transcellular TRPM6 channel along with basolateral Mg^2+^ extrusion of Na^+^/Mg^2+^ exchanger. Inactivating mutations in claudins 16 and 19 in TALH are responsible for familial hypomagnesemia hypercalciuria nephrocalcinosis (FHHNC types I and II). Defective Mg^2+^ reabsorption in the DCT inevitably causes renal hypomagnesemia because there is no Mg^2+^ reabsorption in the downstream DCT. Gene mutations related to the regulation of Mg^2+^ transport in the DCT can cause renal hypomagnesemia. These genes include SLC12A3 encoding thiazide-sensitive NCC, TRMP6 encoding apical TRPM6 channel, HNF1B encoding HNF1β, PCBD1 encoding PCBD1, EGF encoding EGF, EGFR encoding EGFR, KCNJ10 encoding Kir4.1 (EAST syndrome), KCNA1 encoding Kv1.1, FXYD2 encoding γ-subunit of Na^+^-K^+^ ATPase, and CNNM2 encoding CNNM2 (cyclin M2) (Meij et al., 2003; Groenestege et al., 2007; Adalat et al., 2009; Glaudemans et al., 2009; Reichold et al., 2010; de Baaij et al., 2012; Ferrè et al., 2014; Sponder et al., 2016; Chen et al., 2021).

**FIGURE 1:**
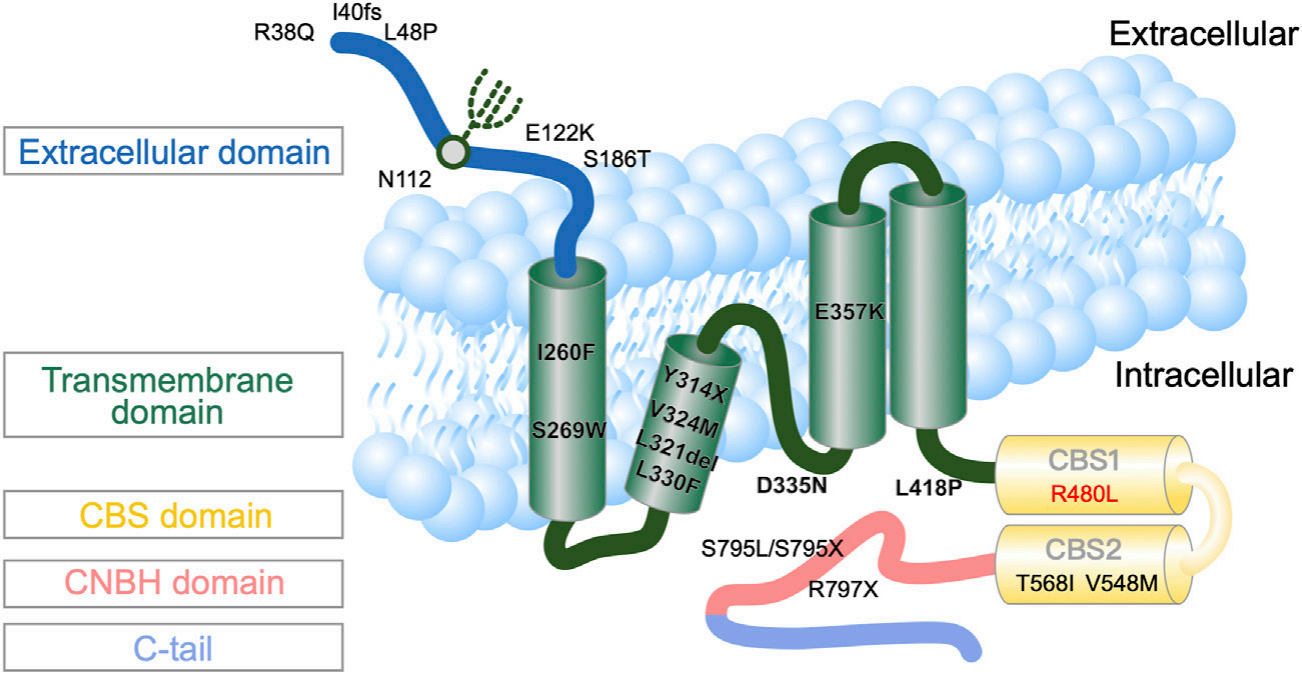
Topography of human CNNM2 with the position of the reported mutations (Stuiver et al., 2011). * indicates the mutation site and location in this patient.

In 2011 and 2014, mutations in CNNM2 predominantly expressed in the DCT and brain were first reported to cause autosomal-dominant (majority) or recessive (minority) renal hypomagnesemia with seizure and intellectual disability (HSMR) (de Baaij et al., 2012; Chen et al., 2021; Franken et al., 2021; Accogli et al., 2019; Arjona and de Baaij, 2018; Bamhraz et al., 2021). In humans, the cyclin M (CNNM, also formerly known as ancient conserved domain protein, ACDP) family has four member proteins (CNNM1–4) sharing evolutionary homology. As shown in Figure 1, the CNNM2 structure consists of an extracellular domain, transmembrane domain (TMD) containing the domain of unknown function-21 (DFU21), Bateman domain or module containing two functionally essential cystathionine β-synthase (CBS) to bind Mg^2+^-ATP, and cytosolic cyclic-nucleotide-binding homology domain (CNBH). To date, only a few families with CNNM2 mutations have been reported (Arjona et al., 2014; Chen et al., 2021; Li et al., 2021). Although CNNM2 was once thought to be a basolateral Mg^2+^ transporter or Na^+^/Mg^2+^ exchanger, more recent evidence points to its function as an intracellular Mg^2+^ sensor, using a conformational change upon binding of Mg^2+^-ATP (Accogli et al., 2019). However, the precise mechanism by which CNNM2 mutation leads to a conformational change and impaired sensing of Mg^2+^-ATP to affect basolateral Mg^2+^ extrusion and/or apical Mg^2+^ reabsorption remains unknown.

We have encountered an infant with refractory hypomagnesemia and excessive renal Mg^2+^ wasting, seizure, and intellectual disability, consistent with the clinical description of HSMR syndrome. In this study, we aimed to identify the genetic mutation for her phenotype and to assess the functional impact of the identified mutation. Our results indicated that a *de novo* heterozygous mutation c.G1439T (R480L) in the CBS domain of the CNNM2 gene was identified in this proband. This missense CNNM2 R480L mutation was found to be pathogenic. *In vitro* studies revealed higher CNNM2-R480L protein expression with proper cellular localization on immunocytochemistry images. The impaired Mg^2+^ efflux with a significant increase in intracellular Mg^2+^ suggests that the CNNM2-R480L mutation blocks Mg^2+^ efflux. In our simulation model, this R480L mutation leads to an attenuated interaction between CNNM2 and ATP-Mg^2+^.

## Materials and Methods

This study was approved by the ethics committee on human studies at Tri-Service General Hospital in Taiwan (IRB2-105-05-136). A trio family including the proband and her parents were enrolled. Written informed consent was obtained from the participants.

### Index Case

The index case was a premature neonate who presented with generalized seizures at the age of 10 days. She was born preterm, gestational age 30 weeks, and was fed with breast and formula milk. No additives to feeding were noticed. The parents were non-consanguineous and no familial history of inherited disorders was reported. Her initial blood pressure was 90/45 mmHg. No anomalies were found on physical examination. Her laboratory characteristics were consistent with hypomagnesemia (0.8 mg/dl) with increased urine Mg^2+^ excretion (FEMg 6.5%), normokalemia, normocalcemia, and normocalciuria (Table 1). There was refractory hypomagnesemia with plasma Mg^2+^ ranging from 0.8 to 1.1 mg/dl despite high-dose oral and intravenous Mg^2+^ supplementation. At her 2 -year-old follow-up, she exhibited delayed neurodevelopment but decreased seizure frequency.

**TABLE 1:** Clinical characteristics.

Characteristics	Patient
Gender	Female
Age at manifestation	10 days
Follow-up	2 years
Symptom at presentation	Seizure
Neurodevelopment	Delayed development milestone
Initial serum Mg^2+^	0.9 mg/dl
FeMg	5.8%
Follow-up serum Mg^2+^	0.8–1.1 mg/dl
Mutation (DNA level)	c.G1439T
Mutation (protein level)	p.R480L
Zygosity	Heterozygous (*de novo*)
Treatment	Levetiravetam
	Magnesium oxide (150 mg/kg/day)

### Whole Exome Sequencing and Direct Sanger Sequencing

Genomic DNA was isolated from a peripheral venous blood sample. We performed exome capture using the Agilent Sure Select v6 and massively parallel sequencing using the HiSeq 4000 platform as previously reported (Li and Durbin, 2009; DePristo et al., 2011). Raw image analyses and base calling were performed using Illumina’s Pipeline with default parameters. Sequence data were aligned to the reference human genome using the Burrows–Wheeler Aligner (BWA) (Li and Durbin, 2009), and duplicate reads were removed using Picard tools. We used the Genome Analysis ToolKit (GATK) to perform the re-alignment and variation (SNP and InDel) detection (DePristo et al., 2011). Annovar was utilized to catalog the detected variations (Wang et al., 2010). Then, we filtered variations with a homo-polymer length >6 (and synonymous substitutions) or that were common (>2%) by dbSNP150 (http://www.ncbi.nlm.nih.gov/projects/SNP/), HapMap, the 1000 Genomes Project (http://www.1000 genomes.org), Exome Aggregation Consortium (ExAC) database, and the Genome Aggregation Database (gnomAD, https://gnomad.broadinstitute.org). Direct Sanger sequencing was performed for all patients and their parents to verify the genetic variants detected by WES. The data that support the findings of this study are available from the corresponding author upon reasonable request.

### Cell Culture, Construction of Plasmids, and Transfection

Murine distal convoluted tubular (mDCT) cells were cultured in a 1:1 mixture of Dulbecco’s modified Eagle’s medium with 1 g/L glucose, 1 mM sodium pyruvate, and Ham’s F-12 Nutrient Mix. Finally, 5% (v/v) fetal bovine serum,100 U/ml penicillin, and 0.1 mg/ml streptomycin were added to the growth medium. The cells were incubated at 37°C in a humidified 5% CO_2_ incubator. Human CNNM2 cDNA (NM_017649.5) was cloned into the pcDNA3.1 vector. The disease-causing mutation was obtained by a QuikChangeTM Site-Directed Mutagenesis Kit (Stratagene, San Diego, CA, United States of America). The primers used to introduce the mutations were R480L: forward primer 5′-GAG​CGG​CTA​CAC​CCT​CAT​TCC​AGT​GTT​TG-3′; reverse primer 5′-CAA​ACA​CTG​GAA​TGA​GGG​TGT​AGC​CGC​TC-3′, R480K: forward primer 5′-GGA​GAG​CGG​CTA​CAC​CAA​GAT​TCC​AGT​GTT​TGA​AGG-3′; reverse primer 5′-CCT​TCA​AAC​ACT​GGA​ATC​TTG​GTG​TAG​CCG​CTC​TCC-3′, V548M: forward primer 5′CTC​ACC​TGG​CTA​TCA​TGC​AGC​GGG​TAA​ACA​ATG-3′; reverse primer 5′-CAT​TGT​TTA​CCC​GCT​GCA​TGA​TAG​CCA​GGT​GAG-3′, T568I: forward primer 5′-GAA​GTT​CTG​GGA​ATC​ATC​TTA​GAA​GAT​GTG​ATT​G-3′; reverse primer 5′-CAA​TCA​CAT​CTT​CTA​AGA​TGA​CGA​TTC​CCA​GAA​CTT​C-3′. The mDCT cells seeded in a six-well plate with 70–90% confluence were transfected by the indicated amount of plasmid DNA with a Lipofectamine 3000 Reagent (Thermo Fisher Scientific). The reported R480K, V548M, and T568I constructs were selected as negative controls. R480K constructed in CBS1 was selected as a charge-unchanged control. Two previously reported mutant constructs (V548M and T568I) in CBS2 were also performed.

### Western Blotting

The mDCT cells were harvested for 24 h after transfection. The cell lysates were prepared in a RIPA lysis buffer with a protease inhibitor cocktail (Roche). Following the separation by centrifugation, the remaining protein lysates were denatured in an SDS sample reagent with 100 mM DTT for 30 min at 37°C and then analyzed by polyacrylamide-SDS mini-gels. The mDCT cells were transfected with a pcDN3.1 empty vector or disease-causing mutation and harvested after 24 h. The cell membrane fraction was subjected to the ProteoExtract native membrane protein extraction kit (Merck-Millipore), following the manufacturer’s description, and then analyzed by semiquantitative immunoblotting (Yamazaki et al., 2013; Chen et al., 2018). The immunoblots were detected with specific antibodies: self-generated CNNM2 (X1000), CNNM2 (X200 Cusabio), α-actin (X30000; Santa Cruz), Hsp70 (X2000; Enzo Life Science), and Na^+^-K^+^ ATPase (X1000; Santa Cruz).

### Immunocytochemistry

The mDCT cells were seeded on a chamber slide (Millicell EZ slide) and transiently transfected with 0.5 μg of plasmid DNA. After 24 h , the cells were washed with PBS and fixed with 4% paraformaldehyde for 15 min. After PBS rinses, the cells were incubated for 1 h with 0.1 Triton X100 in PBS and then blocked with 1% BSA in PBST for another 30 min. Specific antibody CNNM2 (X200 Cusabio) was used for cell staining. After PBST rinses, cells were incubated with Alexa Fluor 488-conjugated goat anti-rabbit (X200 Invitrogen) for 1 h and stained with DAPI (5 ug/ml) for 5 min. The images were captured with a Leica DM2500 microscope.

### Mg^2+^-Efflux Assay

The mDCT cells were cultured on a 96-black, clear bottom tissue culture plate (Corning), after transfection (24 h). The Mg^2+^ imaging of transfected cells was analyzed with Magnesium Green™ (Molecular Probes) as described previously (Yamazaki et al., 2013; Chen et al., 2018), with slight modifications. The cells were incubated with Mg^2+^ loading buffer including 2 μM Magnesium Green (Molecular Probes) at 37°C for 60 min. Then, the buffer was changed to buffer without Mg^2+^ (MgCl_2_ was replaced with 60 mM NaCl) and the fluorescent was recorded at 1-min intervals. The cells images were detected by ImageXpress Micro XLS (molecular devices) and fluorescence was measured using MetaXpress High content image acquisition and analysis software (Molecular Devices). The cell fluorescence was analyzed by the software setting for Cell Scoring.

### Simulation of the Mutant Models

The resolved structures of the human CNNM-PRL complex (PDB code: 5LXQ) (Meij et al., 2003) were used as a template to generate the R480L mutant using the Built Mutants protocol (Biovia Discovery Studio 2019). The CHARMm force field was applied to the model followed by energy minimization using the smart algorithm in the calculation.

### Statistical Analysis

The results were presented as mean ± standard deviation (SD) for continuous variables. Student’s *t*-test was used to compare differences between groups. When comparing the ratio of differences between groups, we used a ratio paired *t*-test with the Holm–Šídák method. A *p*-value less than 0.05 was considered statistically significant.

## Results

### Identification of Novel *CNNM2* Mutation

**FIGURE 2:**
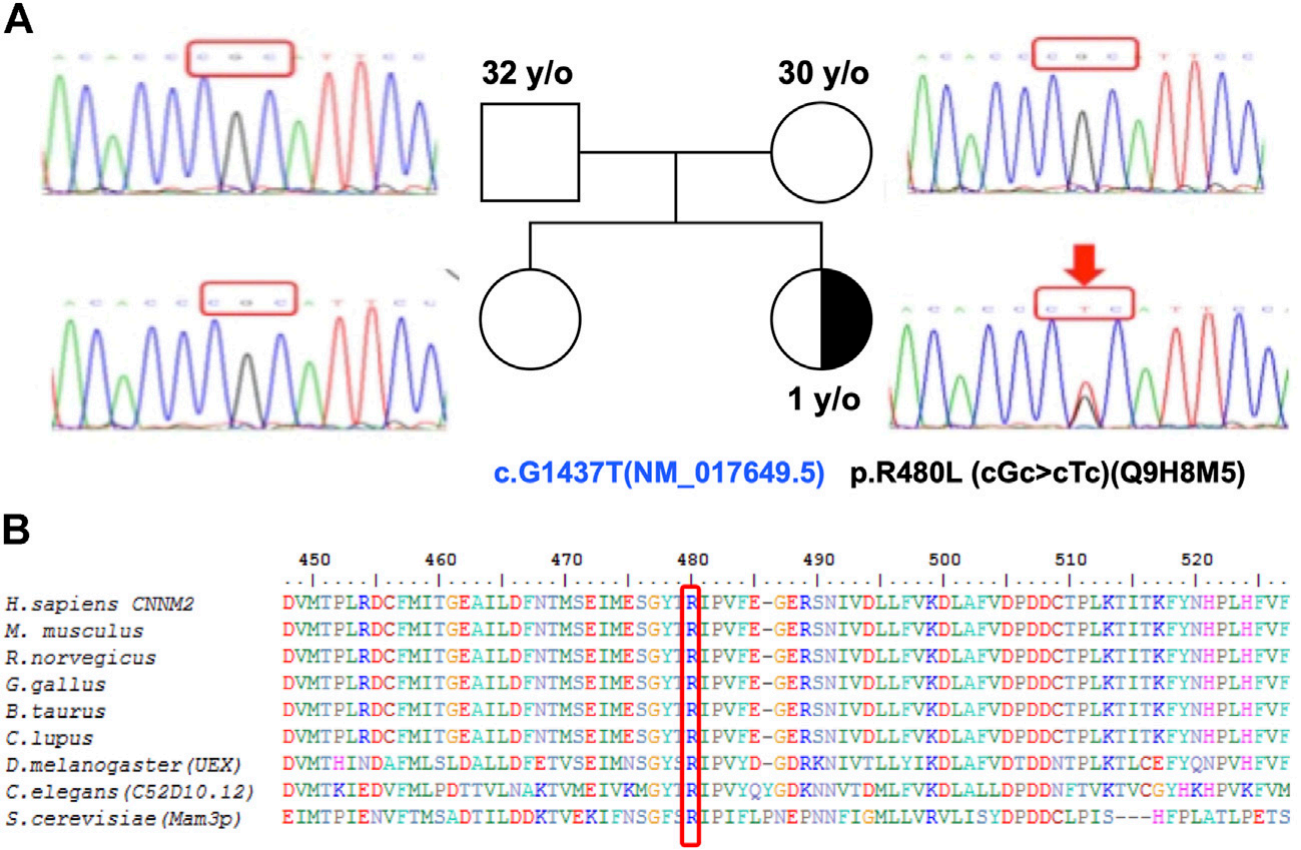
A multiple sequence alignment shows R480 as a highly conserved residue. **(A)** Pedigree showing the index patient and her parents with heterozygous CNNM2 mutation with a half-filled black symbol. **(B)** The R480 of CNNM2 is a highly conserved amino acid (Stuiver et al., 2011). The sequence accession IDs are *H. sapiens*: Q9H8M5, *M. musculus*: Q3TWN3, *R. norvegicus*: Q5U2P1, *G. gallus*: K7CZV7, *B. taurus*: A0A3Q1LRB9, *C. lupus*: E2RJ19, *D. melanogaster (UEX)*: A0A0B7P9G0, *C. elegans (C52D10.12)*: A3QM97, and *S. cerevisiae (Mam3p)*: N1NX85.

To complete genetic diagnosis and counseling for this family, we pursued whole exome sequencing for the patient to identify the cause of pathogenesis. We obtained an average of 6.3G bases mapped to target exon regions with a mean depth of 81 times. About 98.18% of exons were covered at least 10 times. Overall, 53,172 single nucleotide variants and 6,224 small insertions or deletions were identified. Whole exome sequencing did not reveal mutations in SLC12A3, CLDN16, CLDN19, CLCNKB, and KCNJ10 but revealed one novel missense mutation (c.G1439T, p.R480L) located on the cystathionine-β-synthase (CBS) domain of CNNM2. Sanger sequencing for the patient and her parents confirmed this *de novo* heterozygous mutation in the *CNNM2* gene (Figure 2A). R480 is a highly conserved amino acid in different animal species as shown in Figure 2B.

### *In vitro* Expression of Novel CNNM2 Mutants

**FIGURE 3:**
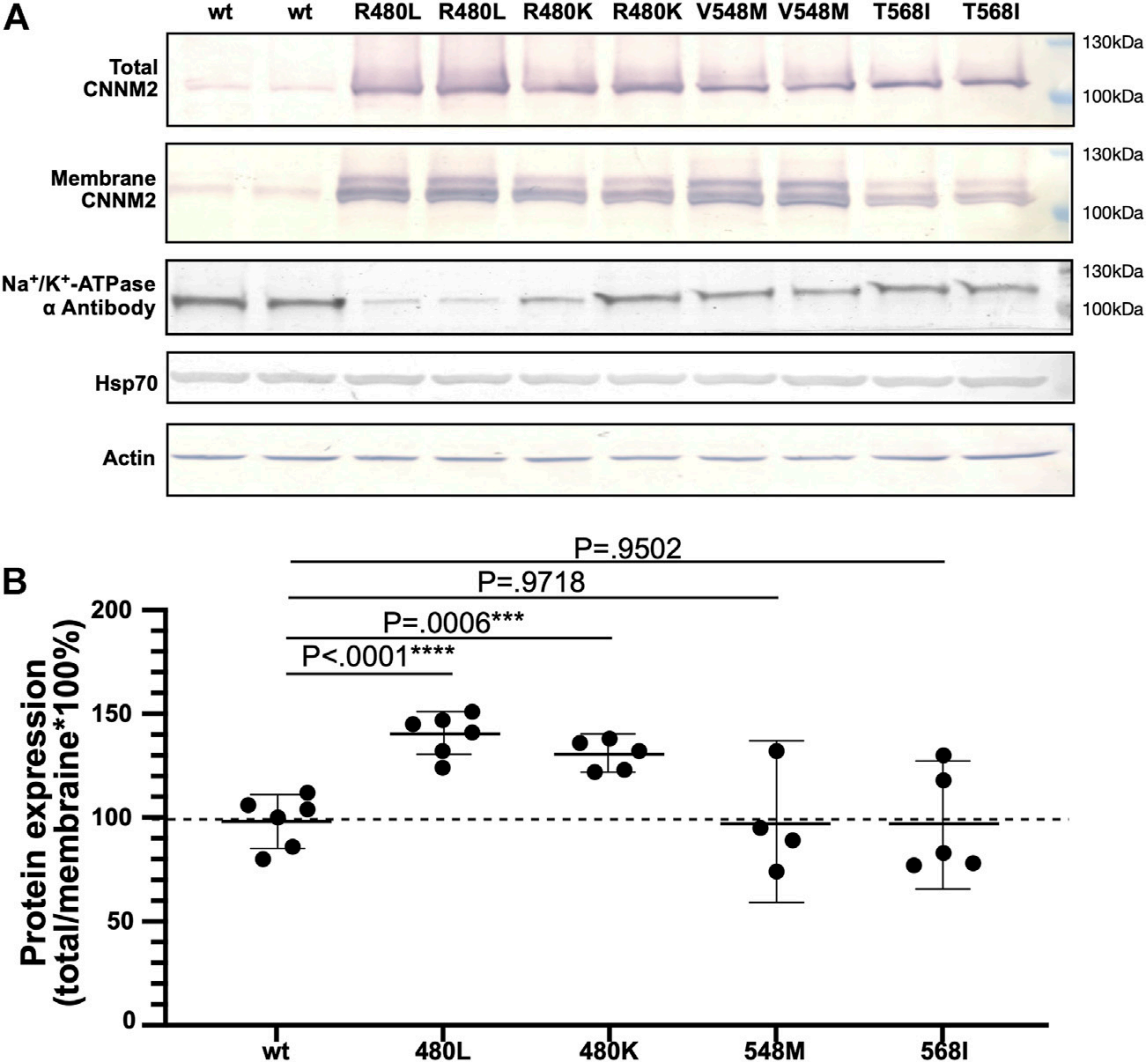
Expression of the mutant CNNM2. **(A)** mDCT cells were transfected with pcDNA3.1.HA.CNNM2-wild type, pcDNA3.1.HA.CNNM2-R480L, pcDNA3.1.HA.CNNM2-R480L, pcDNA3.1.HA.CNNM2-V548M, and pcDNA3.1.HA.CNNM2-T568I expression constructs. Cells extracts were subjected to western blot analysis and probed with antibodies against CNNM2. Antibodies to α-actin and Hsp70 were for loading control of mDCT cells. **(B)** Results are representative of three independent experiments and show increased membrane expression of CNNM2-R480L and CNNM2-R480K.

The total and membrane expressions of wild-type CNNM2 (amino acids 1–875) and mutant CNNM2 (R480L, R480K, V548M, and T568I) proteins were examined after transient transfection of CNNM2 in mDCT cell lines. As shown in Figure 3 and Supplementary Table S1, the membrane expression was increased in CNNM2-R480L and CNNM2-R480K but not in CNNM2-V548M and CNNM2-T568I. Actin and HSp70 were used as the loading control and ubiquitination, respectively. There was no difference in the expression of actin and Hsp70 between cells with wild-type and all CNNM2 mutants.

### Immunocytochemistry Microscopy

**FIGURE 4:**
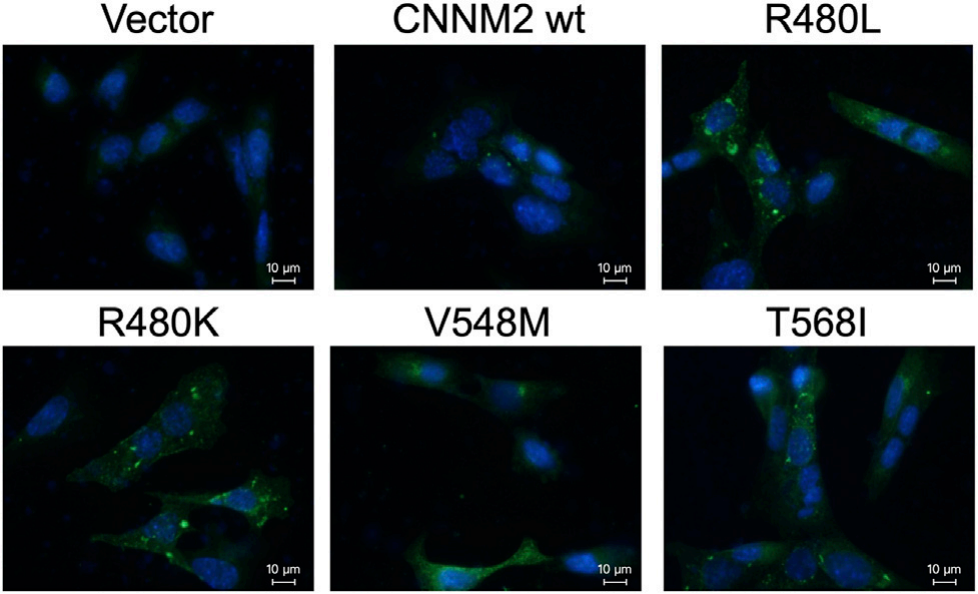
Immunocytochemistry microscopy. Images of transiently transfected murine distal convoluted tubular cells (mDCT) with wild-type and mutant constructs. Cells were immunostained with antibodies against the CNNM2 (in green). Nuclei were counterstained with DAPI (in blue). This demonstrates that the expressions of mutant CNNM2 are higher than those of wild-type CNNM2.

The immunocytochemistry images of mDCT cells with anti-nuclei (blue) and anti-CNNM2 (green) demonstrated that CNNM2-wild type, CNNM2-R480L, and other negative controls (CNNM2-V548M and CNNM2-T568I) were properly localized adjacent to the cell membrane (Figure 4). Of note, the expression of CNNM2-R480L was higher than that of the CNNM2-wild type.

### Mg^2+^-Efflux Assays

**FIGURE 5:**
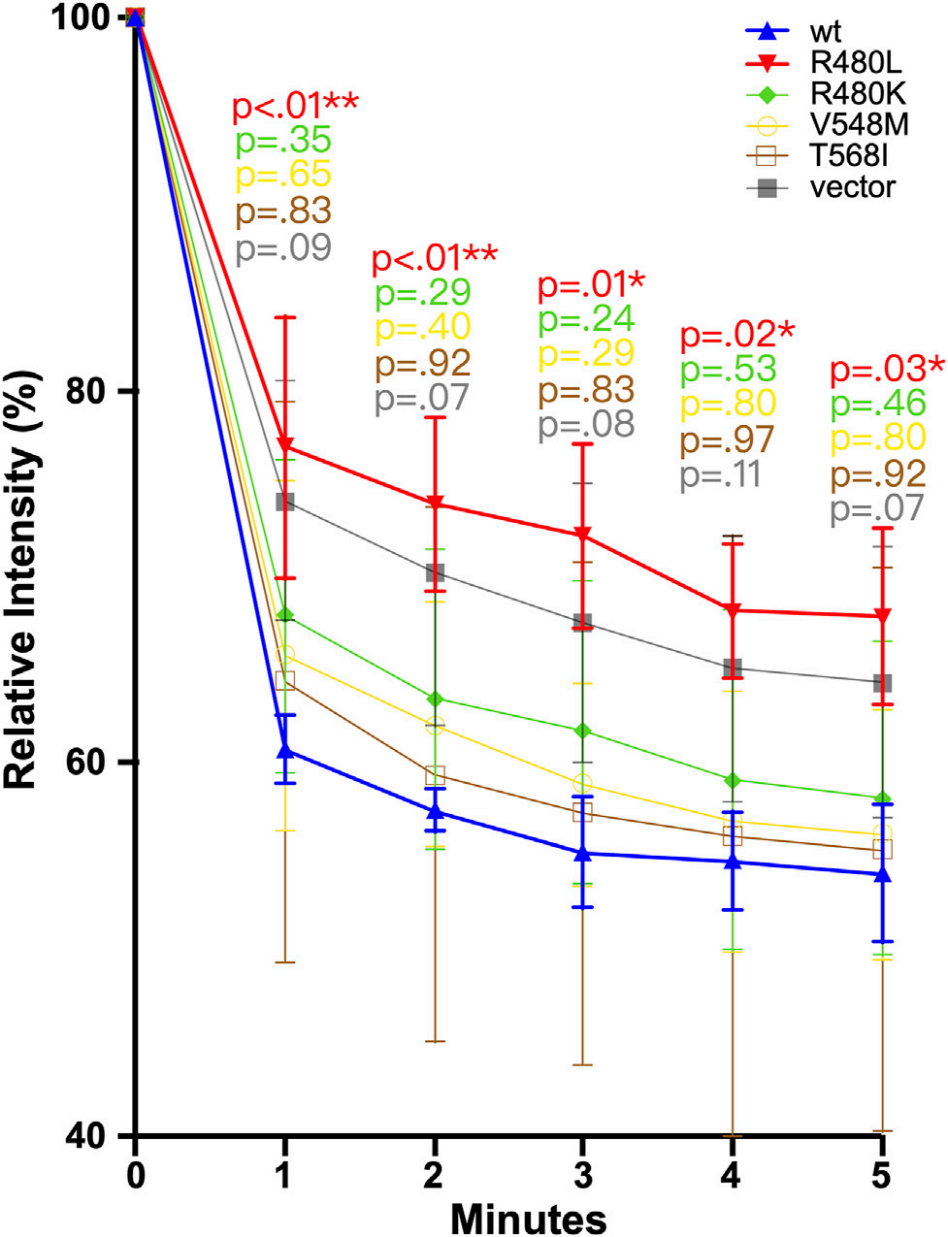
Magnesium efflux assay. mDCT cells transfected with the indicated constructs were loaded with Mg^2+^ green and then subjected to the Mg depletion condition. R480L had significantly reduced Mg^2+^ efflux than other mutants and wild type. In comparison with wild-type and mutant, a *p*-value was calculated. A *p*-value <0.05 is considered statistically significant. *p<0.05, **p < 0.01.

To evaluate the impact of R480L on the function of CNNM2, we examined the CNNM2-dependent Mg^2+^ efflux in a cellular assay with wild-type and mutant CNNM2. The mDCT cells were transfected with the indicated constructs treated with Mg^2+^ Green and then subjected to Mg^2+^ depletion. As shown in Figure 5 and Supplementary Table S2, intracellular Mg^2+^ Green was significantly higher in CNNM2-R480L compared to wild type and other CNNM2 mutants, with *p*-values < 0.05 from min 1 to min 5. This finding indicates that the R480L mutation located in the CBS1 domain of CNNM2 blocks Mg^2+^ efflux activity.

### Simulation Model of the CNNM2 Mutant

**FIGURE 6:**
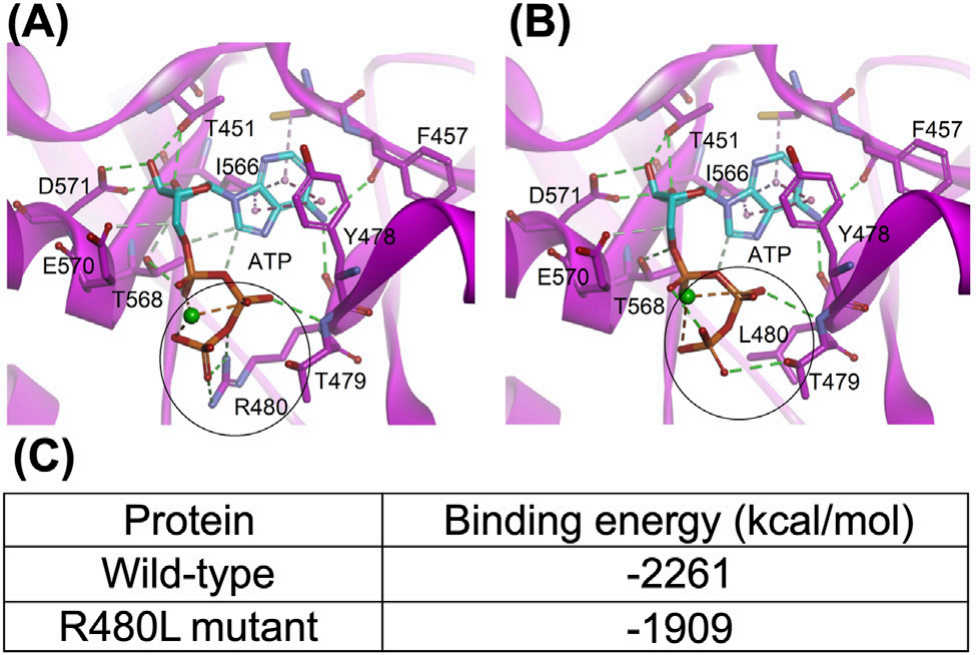
Structure of the CNNM-PRL complex (PDB code: 5LXQ). **(A)** and **(B)**, the wild-type and R480L mutant CBS module of CNNM2, respectively. The dashed lines represent the hydrogen bond (green), electrostatic (orange), and hydrophobic (pink) interactions. The metal ion is shown as a green sphere. The black circle highlights the mutation site; **(C)** indicates that this mutation in the CBS module results in significant impairment of the binding ability with MgATP.

The interaction from the side chain of Arg480 with the γ-phosphate of ATP was lost after being replaced by leucine (Figure 6). The mutation of arginine to leucine led to the reduction of the binding energy of Mg^2+^-ATP in the CBS module of CNNM2 by approximately 352 kcal/mol, which suggested that this mutation may cause significant impairment in the binding ability with Mg^2+^-ATP.

## Discussion

In this study, we have identified a novel and *de novo* heterozygous mutation c.G1439T (R480L) in the CBS domain of the *CNNM2* gene in a trio family with typical HSMR. *In vitro* studies showed that this CNNM2-R480L had a higher expression level than the CNNM2-wild type and proper localization to the plasma membrane. The Mg^2+^ efflux assay in mDCT cells revealed the blockade of intracellular Mg^2+^ efflux under Mg^2+^ depletion. The simulation model also predicted the attenuated interaction of this mutant protein with Mg^2+^-ATP.

To date, nearly 20mutations including ours in *CNNM2* have been reported responsible for HSMR (Stuiver et al., 2011; de Baaij et al., 2012; Arjona and de Baaij, 2018; Accogli et al., 2019; Bamhraz et al., 2021; Franken et al., 2021). Autosomal-dominant inheritance is the most common type with the majority of mutations being *de novo* and missense as shown in this patient (Stuiver et al., 2011; de Baaij et al., 2012; Accogli et al., 2019; García-Castaño et al., 2020; Chen et al., 2021; Franken et al., 2021). The clinical features including profound hypomagnesemia, epilepsy, and neurodevelopmental delay in our proband were in line with phenotypes reported by a previous large cohort study (Franken et al., 2021).) Epilepsy might be a result of disturbed brain development rather than impaired Mg^2+^ efflux. Arjona *et al.* had shown that knockdown of CNNM2 isoforms in zebrafish resulted in disturbed brain development including neurodevelopmental impairments (Arjona et al., 2014). Although the correlation between phenotype and genotype was poor, patients with homozygous mutation usually have more severe forms of structural brain abnormalities (Stuiver et al., 2011; Accogli et al., 2019; Franken et al., 2021).

R480L mutation involved a highly conserved residue in the CBS domain of CNNM2 protein and was pathogenic based on a higher score of pathogenicity by different software prediction and ACME criteria (Richards et al., 2015). We first examined *in vitro* studies for this R480L mutant. A higher CNNM2-R480L protein expression with proper cellular localization on immunocytochemistry images was found. In contrast to the previous findings, the membranous expressions of mutant CNNM2-R480L were higher than those of the CNNM2-wild type (Franken et al., 2021). The exact mechanism of increased expression of CNNM2-R480L on the plasma membrane remained to be elucidated. We speculate that it is secondary to a compensatory effect of impaired function of mutant or different unique mutations in the CBS domain. We confirmed that the R480L mutation did not alter protein trafficking or membrane localization. Both arginine (R) and lysine (K) are basic amino acids with similar physiochemical properties. The difference between the membranous expression of R480L and R480K was minimal in this study. However, the charged R containing the guanidino head group, as opposed to the amino head group in the charged K, is solvated by more oxygen atoms and can form more H-bonds. However, the charged R containing guanidino head group interacts with individual polar molecules more weakly due to its delocalized charge (Meloni et al., 2020). Furthermore, the positively charged residues R480 in CNNM2 plus the threonine residue of T568 are essential for stabilizing a phosphate moiety (Corral-Rodríguez et al., 2014).

**FIGURE 7:**
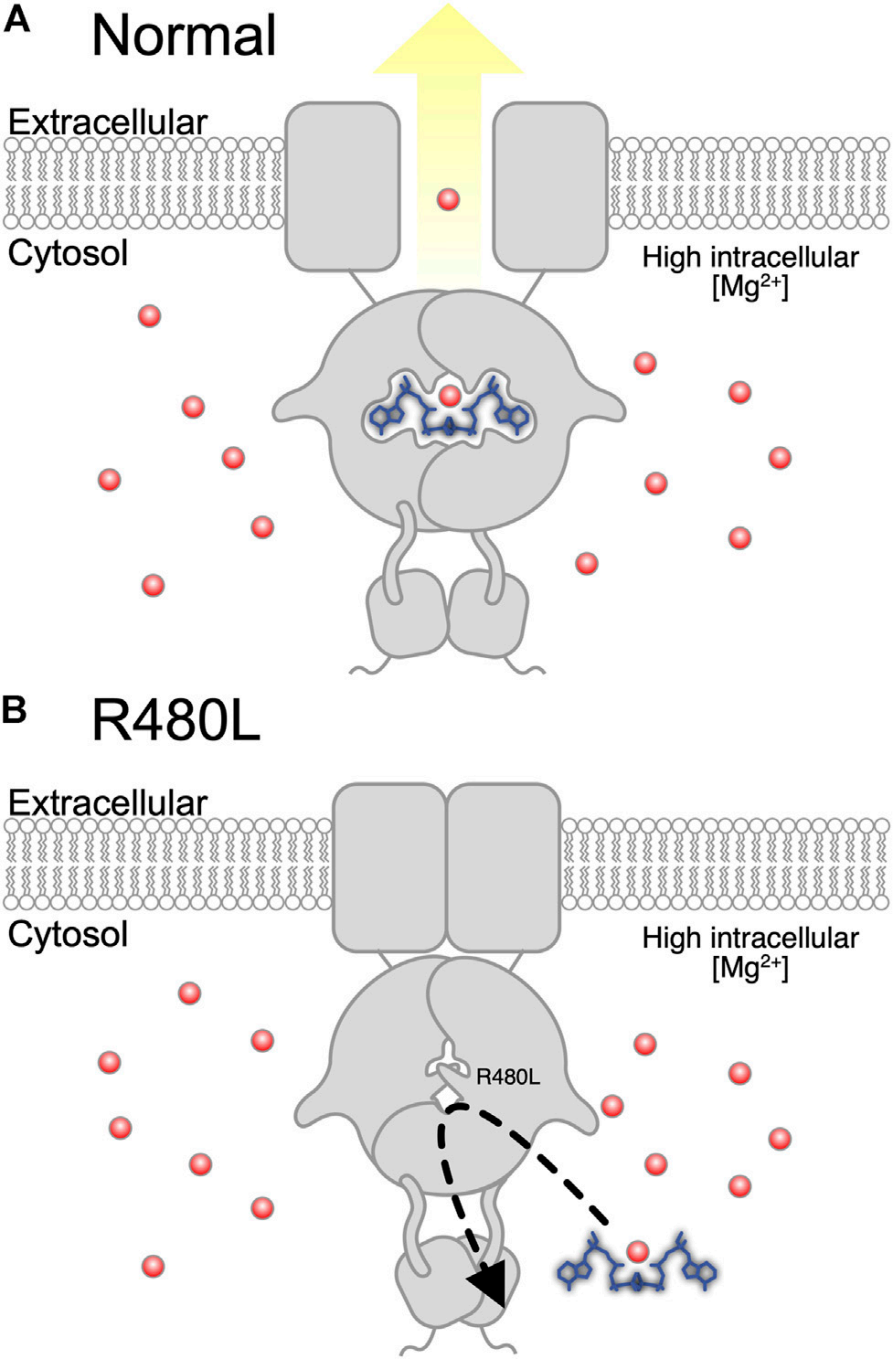
Proposed pathogenesis of hypomagnesemia due to R480L of *CNNM2*. **(A)** Normally, Mg^2+^ is effluxed after Mg^2+^-ATP binds to wild-type CNNM2 under magnesium depletion. **(B)** Mutation in *CNNM2* yields a decreased binding force between CNNM2 and Mg^2+^-ATP and subsequent hypomagnesemia under magnesium depletion.

CNNM2 contains an extracellular N-terminal domain, a transmembrane domain of the unknown function (DUF21), and a large cytosolic region including a cystathionine-β-synthase (CBS) domain and a putative cyclic nucleotide-binding homology (CNBH) domain (Chen et al., 2020). The mutation R480L is located in the CBS domain, and this Arg480 has been reported to interact with Glu570 to form a salt bridge and with the acidic aspartate residue D583 for strengthening CBS dimerization (Corral-Rodríguez et al., 2014; Chen et al., 2020). Previous studies have demonstrated the importance of CBS dimerization for binding Mg^2+^-ATP by the mutation of hydrophobic residues lining the interface of dimerization (Baykov et al., 2011; Chen et al., 2020). In line with this finding, our simulation model predicts that R480L alters the binding force between CNNM2 and Mg^2+^-ATP. Therefore, R480L might diminish its binding with Mg^2+^-ATP by impairment of the formation of CBS dimerization (Figure 7).

We conducted a cellular Mg^2+^ efflux assay to evaluate the effect of R480L on CNNM2 transport activity. Consistent with previous studies, R480L impaired the Mg^2+^ efflux activity in the presence of significantly high intracellular Mg^2+^ under Mg^2+^ depletion. It has been shown that Mg^2+^-ATP binding is required for cellular Mg^2+^ efflux since mutations that abolished Mg^2+^-ATP binding prevent Mg^2+^ efflux (Arjona and de Baaij, 2018). Of note, our simulation model predicts the reduction of the binding energy of Mg^2+^-ATP in the CBS1 of CNNM2 by R480L mutation. However, we did not find a significant impairment of Mg^2+^ efflux in CNNM2 T568I in CBS2, unlike a previous report (Corral-Rodríguez et al., 2014; Hirata et al., 2014). This discrepancy might be due to the following reasons. First, the cell line (mDCT cell) we utilized was different from that (HEK293) used by the previous study (Hirata et al., 2014). Second, we presented the relative intensity of intracellular Mg^2+^-green by calculating the mean fluorescence of all cells over 300 gray levels. Hirata *et al.* presented the relative intensity as the mean fluorescence of 10 cells. In fact, we found that the relative intensity of CNNM2 T568I on the Mg^2+^ efflux assay was higher than the wild type, although the difference was not statistically significant. Altogether, our study demonstrated that this R480L mutation resulted in the diminished binding with Mg^2+^-ATP and consequently led to impairment of Mg^2+^ efflux. This defective Mg^2+^ efflux might account for the hypomagnesemia in this patient with *CNNM2* R480L mutation.

There were a few limitations in our study. First, we did not provide direct evidence for the R480L mutation in causing impairment of CBS dimerization by the crystal structure. Second, isothermal titration calorimetry was not performed to confirm the diminishment of the interaction of Mg^2+^-ATP and the CBS-pair. However, the simulation model showed the reduction of the binding energy of ATP-Mg^2+^ in the CBS module of CNNM2 by R480L mutation.

## Conclusion

We identified a novel and *de novo* heterozygous R480L mutation in the CBS domain of the *CNNM2* gene in a trio family with severe HSMR. This highly expressed CNNM2-R480L properly localizes to the plasma membrane but impairs Mg^2+^ efflux likely through the attenuated interaction with Mg^2+^-ATP, resulting in the clinical manifestation of refractory hypomagnesemia.

## Data Availability

The datasets presented in this study can be found in online repositories. The names of the repository/repositories and accession number(s) can be found at https://www.ncbi.nlm.nih.gov/bioproject, accession number: PRJNA851908.
