# Supplementary material for: Novel CNNM2 Mutation Responsible for Autosomal-Dominant Hypomagnesemia With Seizure
Source: Front Genet. 2022 Jun 29;13:875013. doi: 10.3389/fgene.2022.875013 (PMC9277586; doi:10.3389/fgene.2022.875013)
Supplement: Supplementary file 1 [file DataSheet1.docx]

**Table S1.** Raw data of figure 3

| **wt** | **Vector** | **480L** | **480K** | **548M** | **568I** |
| --- | --- | --- | --- | --- | --- |
| 100 | 84 | 145 | 122 |  |  |
| 112 |  | 147 | 138 |  | 83 |
| 106 | 87 | 124 | 132 | 132 | 130 |
| 86 | 84 | 151 | 123 | 89 | 78 |
| 80 | 82 | 132 | 136 | 74 | 77 |
| 104 |  | 141 |  | 95 | 118 |

**Table S2.** Raw data of figure 5

| **Min** | **vector** | | | | |
| --- | --- | --- | --- | --- | --- |
| 0 | 100 | 100 |  | 100 | 100 |
| 1 | 74.02 | 69.2 |  | 83.2 | 69.85 |
| 2 | 69.27 | 62.87 |  | 82.03 | 66.83 |
| 3 | 67.6 | 61.6 |  | 78.13 | 62.81 |
| 4 | 64.25 | 59.92 |  | 75.39 | 60.8 |
| 5 | 62.85 | 60.76 |  | 75 | 58.79 |
| **Min** | **wt** | | | | |
| 0 | 100 |  | 100 | 100 | 100 |
| 1 | 61.25 |  | 59.09 | 59.63 | 63.04 |
| 2 | 58.13 |  | 58.71 | 56.52 | 56.52 |
| 3 | 57.19 |  | 58.33 | 52.8 | 52.61 |
| 4 | 54.69 |  | 58.33 | 52.17 | 53.91 |
| 5 | 52.19 |  | 59.47 | 53.42 | 51.3 |
| **Min** | **R480L** | | | | |
| 0 | 100 | 100 | 100 | 100 | 100 |
| 1 | 71.99 | 88.79 | 76.15 | 76.35 | 71.35 |
| 2 | 70.6 | 81.71 | 71.87 | 74.29 | 70.82 |
| 3 | 68.06 | 80.24 | 68.2 | 71.98 | 72.15 |
| 4 | 64.58 | 73.16 | 64.83 | 69.15 | 68.97 |
| 5 | 63.19 | 74.34 | 63.91 | 66.84 | 70.82 |
| **Min** | **R480K** | | | | |
| 0 | 100 | 100 | 100 | 100 | 100 |
| 1 | 59.93 | 74.49 | 79.21 | 63.53 | 62.27 |
| 2 | 58.43 | 67.49 | 75.74 | 58.82 | 56.82 |
| 3 | 54.68 | 67.9 | 72.77 | 55.88 | 57.27 |
| 4 | 52.06 | 65.02 | 72.28 | 52.94 | 53.18 |
| 5 | 51.31 | 65.02 | 69.31 | 51.76 | 53.18 |
| **Min** | **V548M** | | | | |
| 0 | 100 | 100 | 100 | 100 | 100 |
| 1 | 66.83 | 71.27 | 74.92 | 65.56 | 50.31 |
| 2 | 63.88 | 66.31 | 66.47 | 62.91 | 50.63 |
| 3 | 61.43 | 62.63 | 62.24 | 58.28 | 49.69 |
| 4 | 61.43 | 59.4 | 61.33 | 57.62 | 44.69 |
| 5 | 60.93 | 59.18 | 58.01 | 58.28 | 44.38 |
| **Min** | **T568I** | | | | |
| 0 |  |  | 100 | 100 | 100 |
| 1 |  |  | 76.72 | 68.75 | 47.64 |
| 2 |  |  | 70.82 | 64.14 | 43.24 |
| 3 |  |  | 68.2 | 61.51 | 42.23 |
| 4 |  |  | 69.18 | 60.86 | 38.18 |
| 5 |  |  | 66.89 | 60.86 | 38.18 |
